# Association Between LGB Sexual Orientation and Depression Mediated by Negative Social Media Experiences: National Survey Study of US Young Adults

**DOI:** 10.2196/23520

**Published:** 2020-12-03

**Authors:** César G Escobar-Viera, Ariel Shensa, Jaime Sidani, Brian Primack, Michael P Marshal

**Affiliations:** 1 Center for Research on Behavioral Health, Media, and Technology Department of Psychiatry University of Pittsburgh Pittsburgh, PA United States; 2 Department of Physical Therapy University of Pittsburgh Pittsburgh, PA United States; 3 College of Education and Health Professions University of Arkansas Fayetteville, AR United States; 4 Suzanne Dworak-Peck School of Social Work University of Southern California Los Angeles, CA United States

**Keywords:** social media, depression, mental health, sexual minorities, minority stress, GSEM, survey, young adult, adolescent, LGBTQ

## Abstract

**Background:**

Lesbian, gay, and bisexual (LGB) persons are disproportionately affected by depression and have high social media use rates. Negative social media experiences may modify depressive symptoms among LGB persons. We sought to assess the potential influence of negative social media experiences on the association between LGB orientation and depression.

**Objective:**

The aim of this study was to assess the potential influence of negative social media experiences on the association between LGB orientation and depression.

**Methods:**

We performed a web-based survey of a national sample of US young adults aged 18-30 years. We assessed the respondents’ LGB orientation, negative social media experiences, and depression using the 9-item Patient Health Questionnaire. We used generalized structural equation modeling to assess both the direct and indirect effects (via negative social media experiences) of LGB orientation on depression while controlling for relevant demographic and personal characteristics.

**Results:**

We found a conditional indirect effect (ab path) of LGB orientation on depressive symptoms via negative social media experience (a: observed coefficient 0.229; *P*<.001; bias-corrected bootstrapped 95% CI 0.162-0.319, and b: observed coefficient 2.158; *P*<.001; bias-corrected bootstrapped 95% CI 1.840-2.494). The results show that among LGB respondents, for those who reported negative social media experiences in the past year, a 1 unit increase in these experiences was associated with a 0.494 unit increase in depressive symptomatology.

**Conclusions:**

Our results suggest that higher rates of depression among LGB young adults are partially explained by negative social media experiences; these results could help inform future patient/provider conversations about mental health risk and protective factors related to social media use. Reducing these experiences and increasing positive social media experiences among LGB persons may mitigate depressive symptomatology in this population.

## Introduction

Lesbian, gay, and bisexual (LGB) persons are at higher risk of experiencing depression than non-LGB individuals [1-4]. Although the cause of depression is multifactorial, the minority stress model [5] posits that LGB people are exposed to a number of environment- and community-related stressors (eg, discrimination, violence) that interact with person-specific stressors and coping skills to influence mental health outcomes. Interestingly, the dramatic rise of social media use in the last decade has profoundly changed the nature of our social relationships, potentially influencing stressors and coping skills originally described in the minority stress framework. Thus, it is important to understand whether the tenets of the minority stress model generalize to other forms of social stressors, such as those experienced through social media use.

Social media use has been consistently increasing in the United States; approximately 92% of young adults currently report that they have at least one social media account [6,7]. Among LGB individuals, social media websites and mobile apps are now a primary way of connecting with others and building relationships [8,9], sometimes replacing interactions that used to mainly occur face-to-face. LGB young adults may turn to social media to compensate for perceived lack of resources and support in their immediate environment [10]. They may also use social media to learn about their own sexual orientation [11], find LGB-specific resources and services [12], find new friends or partners [13], and find social support [14].

Given the relatively recent growth in social media use in the United States, research conducted on the effects of social media on mental health outcomes found that the content and quality of social media experiences and interactions may be important to our understanding of the roles of social media in myriad mental health outcomes [15-18]. For LGB persons, negative social media experiences may play a critical role in depression disparities. These experiences may include exchanges that make individuals feel sad, depressed, or angry, or viewing content that negatively influences their emotional status. Despite their mostly positive motivations for using social media, a content analysis found that LGB individuals reported a higher frequency of negative effects of social media experiences on their well-being than non-LGB persons [19].

Assessing if and to what extent negative social media experiences are associated with depressive symptoms among LGB young adults may help identify intervention points for potential ways to improve or prevent worsening of depressive symptoms. It may also provide initial evidence that can enable mental health professionals to raise awareness among their patients about the importance of monitoring and improving their social media experiences. Therefore, this research used cross-sectional data from a national web-based survey of US young adults to examine (1) differences in depressive symptoms between LGB and heterosexual young adults and (2) determine whether negative social media experiences partially explain LGB depression disparities.

## Methods

### Participants and Procedures

In March 2018, we commissioned Qualtrics Sample Services to recruit a national sample of US adults aged 18-30 years. Recruitment sought to reflect the sociodemographic characteristics of the 2010 US Census. A total of 2408 study participants responded to a web-based survey developed using Qualtrics Online Surveys. The survey was active for 30 days after invitations were sent. Respondents received points that were redeemable for incentives of their choice, such as gift cards or charitable contributions. Qualtrics Sample Services, in conjunction with the research team, conducted a series of procedures to enhance data quality. First, the survey instrument was pilot-tested with a sample of 30 individuals who were not included in the final study sample to ensure that skip patterns were working correctly and to review data for inconsistencies. Next, a “soft launch” was conducted, during which 10% of the target sample (n=240) completed the survey. Again, the data were reviewed for inconsistencies and to ensure that quotas were being reached. Finally, as the final study sample was being recruited, data were reviewed to remove straight-liners, speeders, and responses with duplicate IP addresses. A total of 94 responses were removed due to low quality data based on these procedures. All study procedures were approved by the University of Pittsburgh Institutional Review Board.

### Measures

#### Demographics

Sociodemographic characteristics included assigned sex at birth, age, highest education level achieved, relationship status, and living arrangement. These factors were included because they may be associated with different measures of social media use and depressive symptoms [16,20]. Assigned sex at birth was assessed as female or male. Age was measured in years. Educational level was assessed with five categories: some high school, high school graduate, some college or technical institute, college graduate, and graduate school. For these analyses, the categories were collapsed into “some college or less” and “college graduate or more.” We used three categories to assess relationship status: single, member of an unmarried couple, and married. Living arrangement assessed the people with whom the respondent was living at the time of the survey, and it included four categories: by myself, parent or guardian, significant other, and other arrangement.

#### LGB Orientation

Self-reported sexual minority orientation was assessed with the single item “Do you consider yourself to be?” and the following options: exclusively straight/heterosexual, mostly straight/heterosexual, bisexual, mostly gay or lesbian, exclusively gay or lesbian, and queer. This item has been extensively used in the literature and is suggested as a best practice for population surveys [21,22]. For this analysis, respondents who described themselves as exclusively straight/heterosexual and mostly straight/heterosexual were categorized as non-LGB, and the remaining four groups were categorized as LGB.

#### Depression

The 9-item Patient Health Questionnaire (PHQ-9) was used to assess depressive symptoms over the last 14 days [23]. Items were scored using a 4-point Likert-type scale with scores ranging from 0 to 3 (“not at all” to “nearly every day”). We summed the scores for all responses and created a continuous scale ranging from 0-27.

#### Social Media Use Time

We asked respondents to provide an estimate, in hours and minutes, of the amount of time they used social media for personal use unrelated to work during a single day. The response options included 0 to 23 hours and 0, 15, 30, and 45 minutes [24,25]. For this analysis, the responses were combined to create a composite, continuous single measure of social media time use.

#### Negative Social Media Experiences

We used four items to assess negative social media experiences. These were adapted from prior research that assessed sexual minority victimization [26,27]. Respondents were asked: “Please think about your experiences on social media over the past year. How often: (a) were you called out or hurt by one of your social media contacts/friends? (b) have you posted something and received negative feedback? (c) have you posted something and received no feedback at all? and (d) have you seen posts or pictures that made you realize you were not invited to a peer's activity/party?” Response options used a Likert-type scale that included 1, never; 2, once or twice; 3, a few times a month; 4, about once a week, and 5, more often. Scores were summed and averaged, generating a continuous score from 1 to 5. Given that these items were adapted for this study, we conducted further principal component and reliability analysis prior to model inclusion.

### Data Analysis

Our initial sample (N=2408) was described using mean (SD) for variables measured on a continuous scale and n (%) for variables measured on a categorical scale. To assess if negative social media experiences, depression, and all covariates varied by LGB orientation (yes/no), we used one-way analysis of variance (ANOVA) for continuous variables and chi-square tests for categorical variables.

While previous research on victimization of LGB individuals [26,27] informed the construct of negative social media experiences, we conducted a principal component analysis (PCA) to assess the underlying factor structure of our particular set of adapted items. The PCA of the negative social media experiences scale helped determine a single-factor structure. This factor accounted for 66% of the total variance observed, with an eigenvalue of 2.62. Factor loadings ranged from 0.86 (for “Were you called out or hurt by one of your social media contacts/friends?”) to 0.77 (for “Have you seen posts or pictures that made you realize you were not invited to a peer's activity/party?”). The Cronbach alpha of .81 showed strong reliability of the scale overall.

After completing these initial steps, we used generalized structural equation modeling (GSEM) to construct two path models. The first model assessed a conditional direct path between LGB orientation and depressive symptoms while controlling for sociodemographic factors and social media time use. Among categorical control variables, referent groups were selected based on groups that are less likely to report depressive symptoms (eg, people who are married, are living with their significant other, and have a higher educational level). Then, we fitted a second path model, constructing a path between LGB orientation and negative social media experience as well as between negative social media experiences and depressive symptoms. For this model, we also controlled for sociodemographic factors and social media time use. We used full information maximum likelihood listwise deletion to account for missing data found within the variables included in our models. This resulted in 2336 responses to fit both the conditional direct and indirect models. Our choice of GSEM was appropriate because GSEM accounts for the Likert-type scales used to assess both depressive symptoms and negative social media experience. Because of the relatively recent use of GSEM to assess mediation paths, we obtained biased-corrected bootstrap estimates and CIs using 200 bootstrapped samples to assess the significance of both direct and indirect path models. Finally, we manually computed the proportion of the total effect that corresponded to both direct and indirect effects of LGB orientation on depressive symptoms. All statistical analyses were conducted using Stata 15 [28].

## Results

Approximately half of our initial sample was female (1223/2408, 50.8%), and the majority were aged 25-30 years (1948/2408, 80.9%) and of White, non-Hispanic race/ethnicity (1607/2408, 68.2%). A total of 497/2408 (20.6%) of the sample reported LGB orientation. The characteristics of the complete sample are presented in Table 1. Depression and negative social media experiences scores differed significantly between LGB and non-LGB individuals (*P*<.001 in both cases), with higher mean scores for LGB participants for both scales. Additionally, time per day on social media, education, and relationship status differed between LGB and non-LGB individuals.

**Table 1:** Characteristics of the sample and associations with LGB orientation (N=2408).

Characteristic		Whole sample	LGB^a^ (n=497, 20.6%)	Non-LGB (n=1911, 79.4%)	*P* value^b^	
Depression, mean (SD)		6.2 (5.6)	8.4 (6.3)	5.6 (5.2)	<.001	
Negative social media experiences, mean (SD)		1.9 (0.8)	2.1 (0.8)	1.8 (0.8)	<.001	
Time per day on social media, mean (SD)		3.1 (2.9)	3.7 (3.1)	2.9 (2.9)	<.001	
**Sex assigned at birth, n (%)^c^**						<.001
	Male	1184 (49.2)	197 (39.6)	987 (51.7)		
	Female	1223 (50.8)	300 (60.4)	923 (48.3)		
**Age (years), n (%)**						.89
	18-24	460 (19.1)	96 (19.3)	364 (19.1)		
	25-30	1948 (80.9)	401 (80.7)	1547 (81.0)		
**Race/ethnicity, n (%)**						.15
	White, non-Hispanic	1607 (68.2)	326 (67.6)	1281 (68.4)		
	Black, non-Hispanic	180 (7.6)	35 (7.3)	145 (7.7)		
	Hispanic	339 (14.4)	84 (17.4)	255 (13.6)		
	Asian	198 (8.4)	32 (6.6)	166 (8.9)		
	Other^d^	32 (1.4)	5 (1.0)	27 (1.4)		
**Education, n (%)**						.002
	Some college or less	1078 (44.8)	252 (50.9)	826 (43.3)		
	College graduate or more	1327 (55.2)	243 (49.1)	1084 (56.8)		
**Relationship status, n (%)**						<.001
	Married	737 (30.6)	111 (22.3)	626 (32.8)		
	Member of unmarried couple	623 (25.9)	167 (33.6)	456 (23.9)		
	Single	1046 (43.5)	219 (44.1)	827 (43.3)		
**Living with, n (%)**						.46
	Significant other	1103 (45.8)	213 (42.9)	890 (46.6)		
	Parent/guardian	505 (21.0)	114 (22.9)	391 (20.5)		
	Alone	444 (18.5)	93 (18.7)	351 (18.4)		
	Other	355 (14.8)	77 (15.5)	278 (14.6)		

^a^LGB: lesbian, gay, and bisexual.

^b^Significance derived from analysis of variance for characteristics measured on a continuous scale and chi-square test for characteristics measured on a categorical scale.

^c^Column percentages may not equal 100 due to rounding.

^d^Included American Indian or Alaskan Native, Native Hawaiian or other Pacific Islander, and multiracial, non-Hispanic.

Our first path c model sought to assess LGB disparities by examining the relationship between LGB orientation and depression while controlling for respondents’ social media time use, sex assigned at birth, age, race, educational level, relationship status, and living arrangement. The results are presented in Table 2. This path c model showed a positive, statistically significant relationship between LGB orientation and higher scores of depressive symptoms, with an observed coefficient of 2.160 (*P*<.001) and a bias-corrected bootstrapped 95% CI of 1.590-2.768.

**Table 2:** Generalized structural equation model (GSEM) results for conditional direct effects of LGB orientation and depressive symptoms (path c model) (N=2336).

Characteristic				Observed coefficient		Bootstrapped standard error	BCB (95% CI)^a^
LGB^b^ orientation				2.159		0.287	1.590 to 2.768
Social media time use				0.286		0.047	0.212 to 0.391
Female sex assigned at birth^c^				0.636		0.227	0.182 to 1.046
Age 18-24 years^d^				–0.025		0.293	–0.587 to 0.548
**Race/ethnicity^e^**							
	Black, non-Hispanic			–0.651		0.408	–1.340 to 0.274
	Hispanic			0.045		0.340	–0.594 to 0.765
	Asian			0.331		0.372	–0.334 to 1.188
	Other			–0.476		0.859	–2.154 to 1.251
**Education level^f^**							
	Some college or less			1.470		0.249	0.908 to 1.968
**Relationship status^g^**							
		Member of unmarried couple	0.257		0.295		–0.371 to 0.822
		Single	0.251		0.411		–0.466 to 1.056
**Living with^h^**							
		Parent/guardian	0.935		0.389		0.306 to 1.834
		Alone	1.002		0.425		0.247 to 1.979
		Other	0.359		0.416		–0.496 to 1.090

^a^Bias-corrected bootstrapped 95% CI (200 bootstrapped samples).

^b^LGB: lesbian, gay, and bisexual.

^c^Reference was Male.

^d^Reference was 25-30 years of age.

^e^Reference was White.

^f^Reference was College graduate or more.

^g^Reference was Married.

^h^Reference was Living with significant other.

In the second path model (Table 3), we sought to determine whether negative social media experiences significantly explained potential higher rates of LGB depression while controlling for the same demographic and personal variables specified in the previous model. We found a statistically significant, conditional indirect effect (ab path) of LGB orientation on depressive symptoms via negative social media experiences (a: observed coefficient 0.229; *P*<.001; bias-corrected bootstrapped 95% CI 0.162-0.319, and b: observed coefficient 2.158; *P*<.001; bias-corrected bootstrapped 95% CI 1.840-2.494).

**Table 3:** Generalized structural equation model (GSEM) results for conditional indirect effects of LGB orientation on depressive symptoms (path ab-c’ model) (N=2336).

Characteristic			Observed coefficient		Bootstrapped standard error		BCB (95% CI)^a^	
**Path a (Negative social media experiences)**								
	LGB^b^ orientation			0.229		0.039		0.162 to 0.319
	Social media time use			0.070		0.008		0.055, 0.084
	Female sex assigned at birth^c^			–0.190		0.030		–0.251 to 0.135
	Age 18 to 24^d^			0.146		0.048		0.057 to 0.251
	**Race/ethnicity^e^**							
		Black, non-Hispanic		0.127		0.076		–0.001 to 0.311
		Hispanic		–0.045		0.049		–0.129 to 0.054
		Asian		0.072		0.059		–0.038 to 0.203
		Other		–0.050		0.145		–0.314 to 0.260
	**Education level^f^**							
		Some college or less		0.071		0.037		0.007 to 0.135
	**Relationship status^g^**							
		Member of unmarried couple		–0.041		0.043		–0.142 to 0.033
		Single		–0.021		0.054		–0.130 to 0.066
	**Living with^h^**							
		Parent/guardian		–0.008		0.058		–0.110 to 0.126
		Alone		0.145		0.062		0.035 to 0.286
		Other		–0.051		0.054		–0.150 to 0.070
		Parent/guardian		–0.008		0.058		–0.110 to 0.126
**Path bc’ (Depressive symptoms)**								
	LGB orientation			1.658		0.314		1.068 to 2.252
	Negative social media experiences			2.158		0.176		1.840 to 2.494
	Social media time use			0.134		0.047		0.054 to 0.233
	Female sex assigned at birth^c^			1.040		0.221		0.691 to 1.536
	Age 18 to 24^d^			–0.359		0.308		–0.943 to 0.189
	**Race/ethnicity^e^**							
		Black, non-Hispanic		–0.856		0.447		–1.635 to 0.183
		Hispanic		0.165		0.352		–0.438 to 1.048
		Asian		0.191		0.362		-0.527 to 1.090
		Other		–0.366		1.064		–2.468 to 1.697
	**Education level^f^**							
		Some college or less		1.326		0.237		0.891 to 1.872
	**Relationship status^g^**							
		Member of unmarried couple		0.342		0.292		–0.190 to 0.878
		Single		0.297		0.379		–0.345 to 1.036
	**Living with^h^**							
		Parent/guardian		0.932		0.388		0.224 to 1.748
		Alone		0.725		0.399		–0.244 to 1.401
		Other		0.467		0.355		–0.119 to 1.355

^a^Bias-corrected bootstrapped 95% CI (200 bootstrapped samples).

^b^LGB: lesbian, gay, and bisexual.

^c^Reference was Male.

^d^Reference was 24-30 years of age.

^e^Reference was White.

^f^Reference was College graduate or more.

^g^Reference was Married.

^h^Reference was Living with significant other.

**Figure 1:**
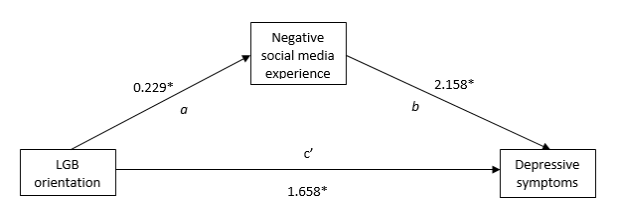
Path model (ab-c’) presenting observed coefficients of the conditional effect of LGB orientation on depressive symptoms via negative social media experiences. LGB: lesbian, gay, and bisexual. *P<.001.

Moreover, the product of multiplying the observed coefficients of paths a and b in our ab-c’ model (Figure 1) showed a conditional indirect effect of 0.494 (approximately 23% of the total effect of LGB orientation on depressive symptomatology), with a direct effect (path c’) equal to 1.658. This indirect effect suggests that among LGB respondents in our national sample of US young adults, and for those who reported negative social media experiences in the past year, a 1 unit increase in these experiences is associated with a 0.494 unit increase in reporting of depressive symptomatology (Figure 1).

## Discussion

### Principal Findings

Sexual minority individuals face well-known mental health disparities, including disparities in depression. In this national web-based survey of US young adults, we found a positive, strong association of LGB orientation with depressive symptoms when controlling for demographic and personal characteristics. We also found that compared to their non-LGB peers, LGB respondents reported significantly more negative social media experiences in the previous 12 months. Importantly, our results suggest that the association of LGB orientation with depression might have an indirect path via this negative social media experiences. Therefore, reducing negative social media experiences among LGB persons may mitigate depressive symptomatology in this population and potentially reduce LGB depression disparities.

### Main Findings

Our analyses showed a direct relationship between LGB orientation and higher PHQ-9 depression scores. These results echo a plethora of previous research that found disparities in depression between this population and their heterosexual peers [4,29,30]. According to the minority stress framework [5], external LGB-related stressors such as social rejection and victimization are associated with more individual-based, proximal stressors (eg, internalized homonegativity), which in turn interact with an individual’s coping skills and social support gathered from the community to buffer the negative impact on their mental health. Interestingly, the defining affordances of social media platforms deeply transformed social interactions. For example, asynchronicity of social media conversations and interactions greatly modifies the ability of the people involved in those interactions to take cues, interpret subtleties, and process the social media experience itself [31]. Therefore, researchers must continue to study the influence of these social media experiences on individuals’ mental health, especially among groups already recognized as mental health disparity populations, such as sexual and gender minority groups (eg, LGB and transgender individuals).

In our national sample of US young adults, we found that LGB respondents had significantly higher scores of negative social media experiences in the previous 12 months compared to non-LGB peers. Our results also showed an indirect relationship between LGB orientation and severity of depressive symptoms via negative social media experiences. While these results confirm previous research reporting higher frequency of web-based discrimination, cyberbullying, and rejection among LGB populations [32], they expand previous findings by identifying a potential mediating role of social media experiences in depression among this group. While one of the items included in our negative social media experiences scale (“How often were you called out or hurt by one of your social media contacts/friends?”) alluded to situations that might occur both on the web or in the physical environment, the other three items (“How often have you posted something and received negative feedback?” “How often have you posted something and received no feedback at all?” and “Have you seen posts or pictures that made you realize you were not invited to a peer's activity/party?”) not only highlight an experience of rejection but also allude to the unique asynchronous nature of the social media experience.

Given our results and the prevalence of depression and social media use among LGB persons, we suggest interventions that seek to empower LGB young people to reduce negative social media experiences or even eliminate experiences these before they happen. Although to our knowledge, these interventions are currently few or nonexistent, results of prior research [33] suggest that sexual and gender minority youth go through a series of decision points while navigating negativity on the web that could be responsive to behavioral interventions. Given the affordances of social media [34], future research must carefully assess a wider array of LGB-specific negative social media experiences to determine whether these experiences represent a distinctive stressor in the minority stress framework. Research should also determine the extent to which the risk posed by social media experiences to the mental health of LGB persons is comparable to the risk posed by discriminatory experiences in the physical environment. For example, permanent accessibility of social media content, as well as the ability to quantify content popularity, may render a seeming “one-time” negative social media interaction more permanent, searchable, shareable, and “likeable” far beyond the original intent. Potential educational interventions could help social media users become familiar with social media account management, vetting of new connections, and use of privacy settings and platform features to protect users’ privacy and make the social media experience less stressful and more enjoyable.

Interventions could also focus on increasing positive social media experiences. This could be achieved by creating and joining supportive social media communities with strong community guidelines, enhancing the ability of LGB youth to make new and safer social media connections, and providing LGB-specific mental health resources, which are among the top needs of LGB youth [35,36]. Prior research has described the importance of synchronous, text-based internet-based interactions for sexual and gender minority youth to find safe, affirming, and supportive spaces on the web that may not exist in their offline lives [37,38]. From a clinical standpoint, although these findings are preliminary, mental health professionals may want to specifically address social media experiences among youth and emerging adults in general and sexual minority persons in particular. Our findings indicate that it may be important to ask about apparently subtle circumstances in which an LGB person may feel ignored and discriminated against (eg, lack of positive reinforcement after posting one’s photograph with a same-sex partner), which could have a lasting impact in an individual’s emotional well-being.

### Limitations

Our study has a number of limitations to consider. While the proposed direction of our analysis was supported by the tenets of the minority stress framework, the lack of longitudinal individual-level data on the variables used in our analysis precludes us from making strong causal inferences from our findings. Moreover, our analyses relied on data from questions that asked participants to report about their social media experiences over a period of 12 months before the survey; because of this, some respondents might have faced limitations when thinking back to the social media experiences they had. However, this is the first study assessing the role of negative social media experiences on mental health outcomes among LGB individuals, a well-known health disparity population. This study could help guide future longitudinal research looking at inter- and intra-individual change over time in both negative social media experiences and depression among LGB populations.

Our conceptualization of negative social media experiences was focused on personal interactions (or the lack thereof), and the questions were not specifically developed to thoroughly assess the components of minority stress. Thus, the items might have not captured the entirety of this construct or its relevance in the broader context of minority stress. While our scale was reliable in assessing negative social media experiences related to interactions between individuals and their social media contacts, we did not assess experiences related to interactions with LGB-specific social media content (eg, LGB-specific negative or discriminatory content within friends’ posts, photographs, or videos posted on individuals’ social media newsfeeds) or the potential differential impact of negative social media experiences on a person-to-person basis compared to a person-to-many (ie, social media groups) basis. However, our results confirm and expand previous research showing higher frequency of negative experiences on the web among LGB persons than among non-LGB individuals. Because of this, and given the unique affordances of social media [31,39], such as asynchronicity and permanence, it is important to further explore these nuances as they apply specifically to the social media experiences of LGB persons and to account for similar experiences in the offline environment.

Although the larger study from which this study was conducted recruited nationally, the respondents’ age skewed toward older young adults, and recruitment did not focus on a nationally representative sample of LGB respondents. Thus, the results of this study are not necessarily generalizable to all groups of young adults or LGB young adults across the United States. Future research in this area should aim to recruit a nationally representative sample focused solely on LGB young adults.

Additionally, we did not assess other social media experiences that may also influence mental health. Previous research has identified a number of social media−related experiences and factors that are associated with positive mental health outcomes among LGB individuals [14,40,41]. These include, but are not limited to, seeking and perceiving the availability of social support on social media, establishing new connections and friendships on the web, and creating network structures of social media friends. Given that negative social media experiences do not occur isolated from other experiences related to social media, future research will benefit from accessing these complex interactions not only longitudinally but potentially also in real time. This will be an important component of understanding the role of social media interactions in LGB individuals’ mental health, specifically depression.

### Conclusions

Our study is part of a growing body of literature focused on analyzing the potential effects of negative social media experiences (one aspect of social media use) on depressive symptoms. The authors’ perspective was one of understanding negative social media experiences, as opposed to previous research that solely focused on the volume of negative social media experience use (ie, amount of time spent on negative social media experiences). This research is critical for two reasons: First, there are no signs that young adults’ negative social media use will decrease in the foreseeable future. Therefore, it is necessary to grasp the subtleties of negative social media experience interactions and their influence on individuals’ mental health. Second, understanding these nuances among minority groups, such as LGB persons, will help inform potential expansion of existing theories that seek to explain mental health disparities within subpopulations of young adults. The results of this study will help inform future patient/provider conversations about mental health risk and protective factors related to negative social media experience use as well as the development of interventions that seek to improve the experiences and mental health of sexual minority populations.

## Abbreviations

LGB: lesbian, gay, and bisexual
GSEM: generalized structured equation model
PHQ-9: 9-item Patient Health Questionnaire
